# Identification of Peptide Mimotope Ligands for Natalizumab

**DOI:** 10.1038/s41598-018-32832-1

**Published:** 2018-09-27

**Authors:** Laura E. Ruff, Jessica A. Pfeilsticker, Nicholas E. Johnsen, Sarah Nocchi, Bradley T. Messmer

**Affiliations:** Abreos Biosciences, 3550 General Atomics Ct, Bldg G02, Rm 137, San Diego, CA 92121 USA

## Abstract

Mimotope peptides selected from combinatorial peptide libraries can be used as capture reagents for immunoassay detection of therapeutic monoclonal antibodies (mAbs). We report the use of phage display libraries to identify peptide ligands (Veritopes^TM^) that bind natalizumab, a therapeutic mAb indicated for use in multiple sclerosis. PKNPSKF is identified as a novel natalizumab-binding motif, and peptides containing this motif demonstrated utility as capture reagents in enzyme-linked immunosorbent assays (ELISAs). A peptide containing the identified motif was shown to be competitive with the natural ligand (α4-integrin) and a neutralizing anti-idiotype antibody for natalizumab binding, indicating that Veritopes^TM^ act as surrogate ligands that bind the antigen binding site of natalizumab. Affinity maturation further confirmed the motif sequence and yielded peptides with greater apparent affinity by ELISA. Veritopes^TM^ are promising assay reagents for therapeutic drug level monitoring.

## Introduction

Multiple sclerosis (MS) is a leading cause of neurologic disability that affects approximately 400,000 people in the US and 2.5 million worldwide^1^. Natalizumab (marketed as Tysabri) is the top selling biologic drug indicated for MS and has been used to treat 177,800 patients worldwide as of November 2017^2^. Natalizumab is highly effective in treating MS patients; in the AFFIRM study, natalizumab was shown to reduce sustained physical disability by 42% relative to placebo^3^. Natalizumab is a humanized recombinant IgG4 mAb that targets the α4 chain of α4β1 integrin (also known as very late activation antigen 4; VLA-4) and a4β7 integrin (LPAM-1). It is thought to function by blocking migration of immune cells across the blood-brain barrier into the central nervous system (CNS), thus suppressing inflammation in patients with relapsing-remitting multiple sclerosis^3,4^.

The immunosuppressive activity of natalizumab has been associated with opportunistic infection by John Cunningham (JC) virus which may lead to progressive multifocal leukoencephalopathy (PML), a serious and often-fatal opportunistic brain infection. Approximately 56–58% of MS patients are positive for anti-JC virus antibodies, which puts them at increased risk for developing PML while on natalizumab^5–7^. The estimated incidence of PML in patients positive for anti-JC virus antibodies is 3.87 per 1,000 patients after at least 1 month of natalizumab treatment. It increases to 11.1 per 1,000 patients for JC virus antibody positive patients with prior use of immunosuppressants and long-term use of natalizumab (24–48 months)^8^. The mortality rate of symptomatic PML patients is 25.8% and most survivors of PML have increased functional disability, measured via EDSS and KPS scores^2^.

The standard dosing regimen for natalizumab is 300 mg by IV infusion every 4 weeks^9^. With standard dosing, serum natalizumab concentrations have been shown to vary over 100-fold^4^. It has been postulated that high serum concentrations may increase the risk of PML^10,11^. Recently, extended interval dosing (infusion every 5–12 weeks instead of every 4 weeks) has been shown to decrease PML risk by 94%^12^. Conversely, low drug concentrations have been associated with a lack of natalizumab efficacy^4^. Multiple enzyme-linked immunosorbent assays (ELISAs) to detect levels of circulating natalizumab have been developed for research purposes^13–15^. A receptor-based flow cytometry assay has also been reported^16^. However, no widely available commercial tests for measurement of blood natalizumab concentrations currently exist.

A process for identifying peptides that bind selectively to therapeutic mAbs as quantitative assay reagents has been previously demonstrated^17^. Here we report the identification of mimotope peptides, termed Veritopes™, that act as surrogate ligands for natalizumab. The performance of Veritopes^TM^ as assay capture reagents for natalizumab is demonstrated by ELISA. Affinity maturation is also performed.

## Results

**Figure 1:**
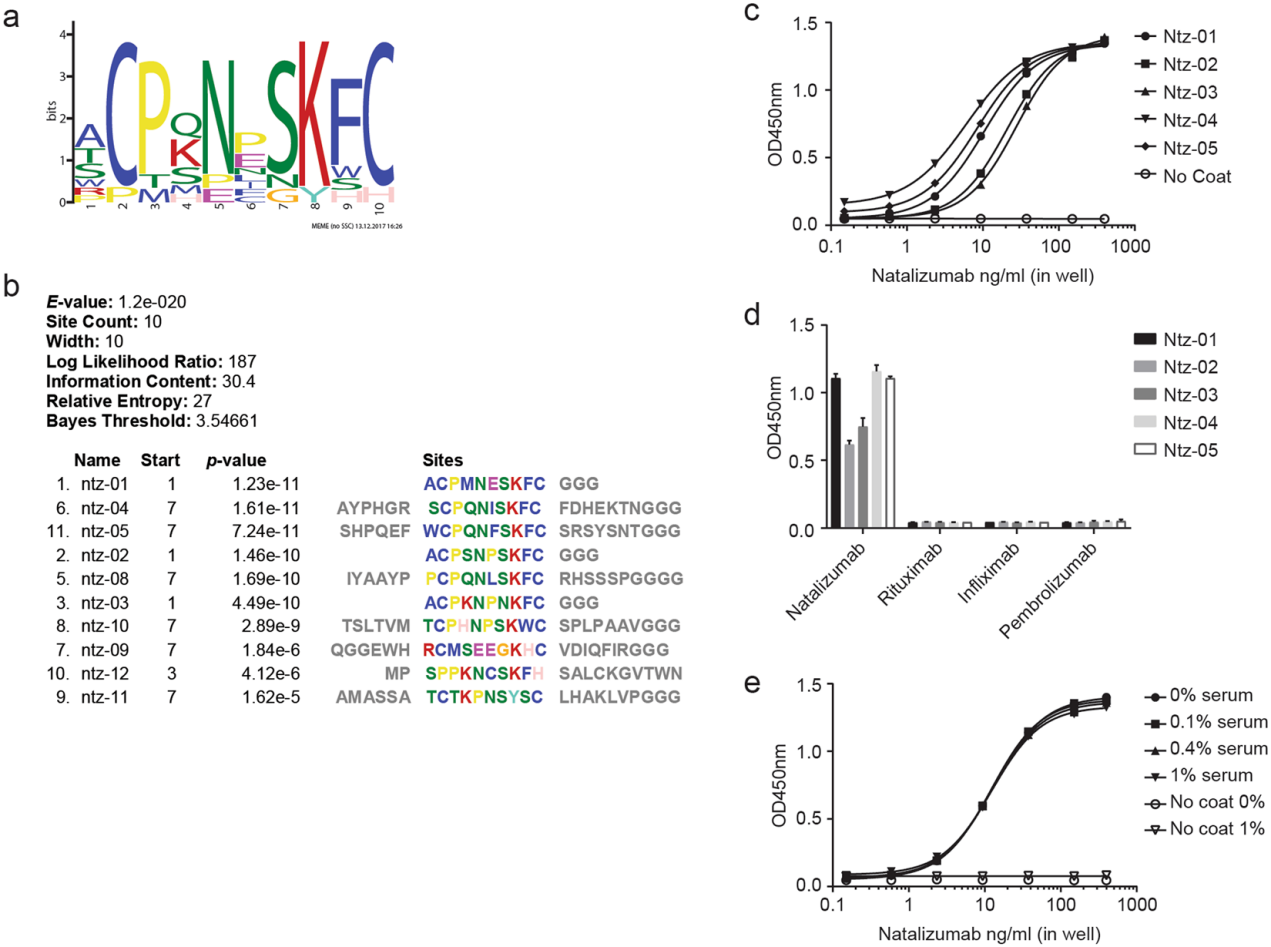
Natalizumab biopanning results and performance of selected peptides in ELISA (**a**) Consensus motif of selected peptide sequences from phage display panning. (**b**) Sequence alignment of motif-containing peptide sequences. (**c**) Natalizumab capture by immobilized synthetic peptides in ELISA. EC50 (ng/ml in well) of calibration curves: Ntz-01: 13.95, Ntz-02: 37.88, Ntz-03: 51.68, Ntz-04: 6.7, Ntz-05: 10.45. (**d**) Selectivity of synthetic peptides against control therapeutic mAbs in ELISA. (**e**) Ntz-01 capture of natalizumab spiked into 0.1–1% human serum. For (**c–e**), one representative experiment is shown, with error bars representing SD of duplicate wells.

Several phage display libraries (Table 1) were biopanned against natalizumab to identify surrogate ligand peptides. Enrichment of binding was noted for three of the selection libraries. Individual phage clones from these selected pools were isolated and sequenced to determine the displayed peptide. For some of the sequenced phage, binding to natalizumab was confirmed with the clonal phage population. There were 11 total natalizumab-binding phage clones, listed in Table 2. A primary sequence motif, shown in Fig. 1a,b, emerged from the cysteine-constrained sequences. Five peptides displaying the motif were synthesized and tested as capture reagents in ELISA on neutravidin- or streptavidin- coated plates. All five peptides bound natalizumab (Fig. 1c) and demonstrated specificity when screened against three control mAb therapeutics (Fig. 1d). Ntz-01 peptide was selected for further testing, due to the highly conserved sequence of Ntz-01, -02, and -03 and lack of adjacent random sequence (as in Ntz-04 and Ntz-05). Additional selectivity of Ntz-01 was confirmed by binding natalizumab in 0.1-1% serum (Fig. 1e). No matrix effects were observed in this serum concentration range.

**Table 1:** Phage display libraries. The forms of phage display libraries used in biopanning. **C** = disulfide bridge.

Library	Sequence
1	XXXXXXX
2	XXXXXXXXXXXX
3	A**C**XXXXXXX**C**
4	XXXXXXX**C**XXXXXXX**C**XXXXXXX

**X** = random. All sequences contained a C-terminal GGG linker as the fusion point of the displayed peptide and the N-terminus of the pIII M13 phage coat protein.

**Table 2:** Selected sequences. Biopanning-selected sequences that were confirmed positive by phage clone binding of natalizumab.

Name	Sequence	Count	Synthesized
Ntz-01	ACPMNESKFC	12/22	Y
Ntz-02	ACPSNPSKFC	6/22	Y
Ntz-03	ACPKNPNKFC	2/22	Y
Ntz-04	AYPHGRSCPQNISKFCFDHEKTN	5/20	Y
Ntz-05	SHPQEFWCPQNFSKFCSRSYSNT	1/20	Y
Ntz-08	IYAAYPPCPQNLSKFCRHSSSPG	6/20	N
Ntz-09	QGGEWHRCMSEEGKHCVDIQFIR	1/20	N
Ntz-10	TSLTVMTCPHNPSKWCSPLPAAV	1/20	N
Ntz-11	AMASSATCTKPNSYSCLHAKLVP	1/20	N
Ntz-12	MPSPPKNCSKFHSALCKGVTWNV	2/20	N
Ntz-13	QTLNHSWLHTFI	1/9	N

The total Count (denominator) includes candidates that were negative by phage clone binding (sequences not shown). All peptides were synthesized with **C**-terminal GGG-Lys(biotin).

**Figure 2:**
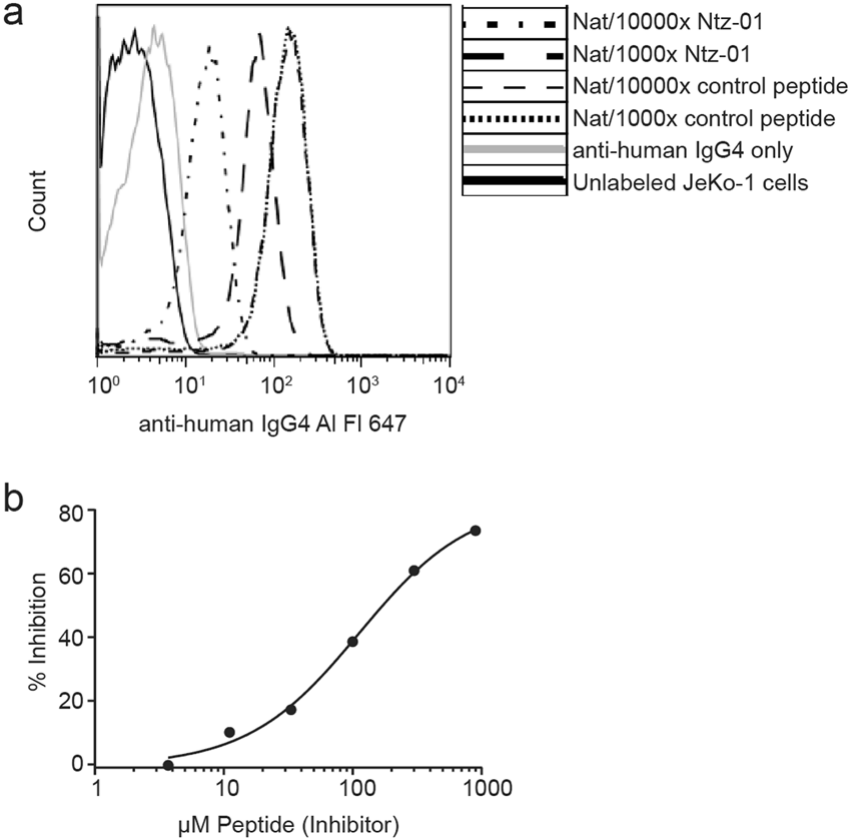
Characterization of Ntz-01 binding epitope (**a**) Ntz-01 competition with CD49d+ JeKo-1 cells for natalizumab binding. (**b**) Ntz-01 inhibition of natalizumab capture by anti-Id ELISA. One representative experiment is shown for each.

The epitope of Ntz-01 was characterized by competition with α4 integrin binding to natalizumab. A series of Ntz-01 concentrations were mixed with natalizumab prior to incubation with CD49d+ (α4 integrin) JeKo-1 cells. Cell-bound natalizumab was detected by fluorescently labeled secondary antibody and quantified by flow cytometry. Concentration dependent Ntz-01 inhibition of natalizumab-cell binding was observed (Fig. 2a). In competition ELISA, Ntz-01 inhibition of natalizumab capture by a neutralizing anti-idiotype (anti-Id) antibody Fab fragment was also observed (Fig. 2b). Confirmation of JeKo-1 cell CD49d expression is shown in Online Resource 1 (Supplemental Fig. 1).

**Figure 3:**
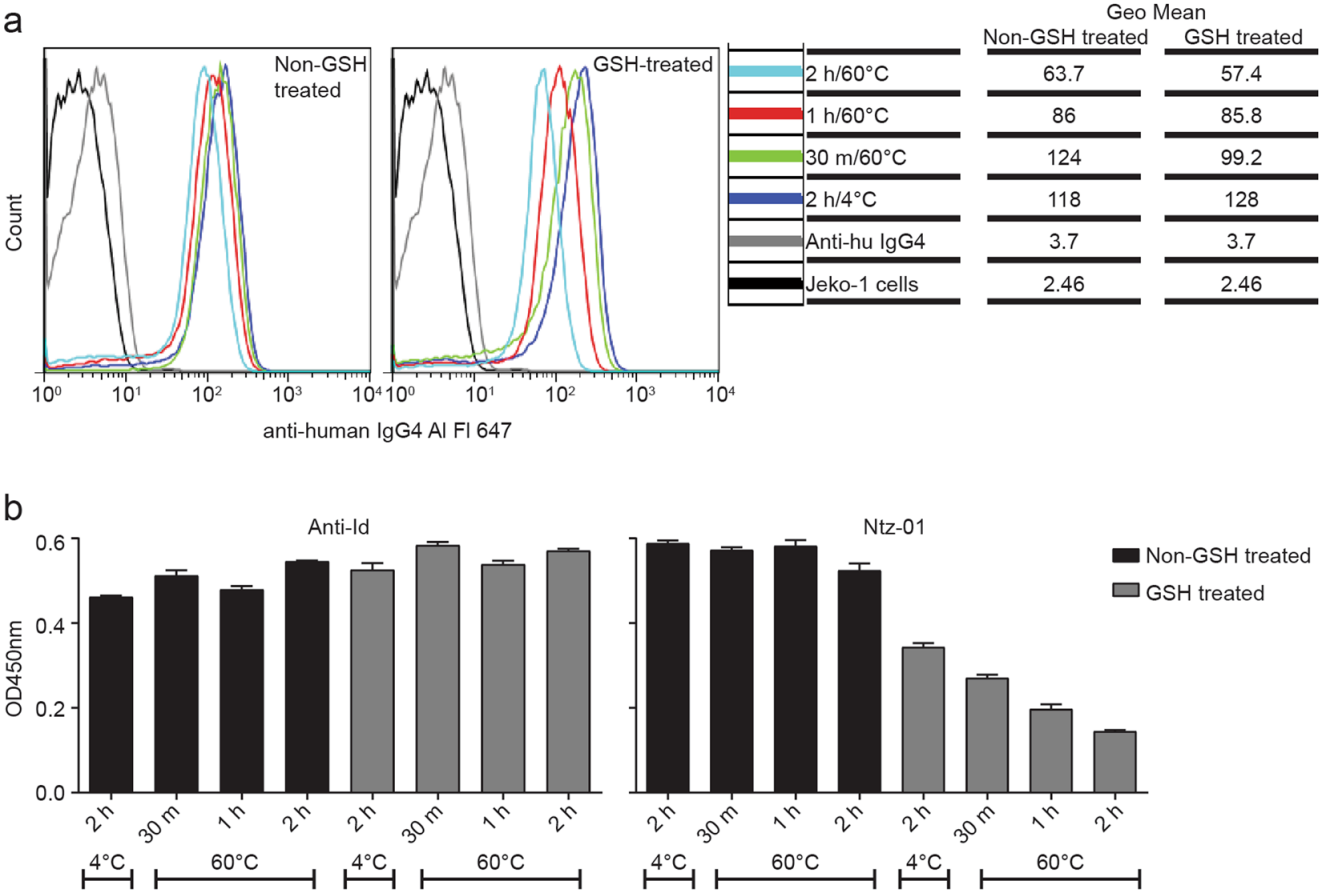
Binding stability of heat exposed natalizumab (**a**) Functional binding of heat exposed natalizumab samples to CD49+ JeKo-1 cells. Both GSH treated and non-GSH treated natalizumab samples were tested. (**b**) Binding of heat exposed natalizumab samples in peptide (Ntz-01) ELISA and anti-Id ELISA. For (**b**) one representative experiment is shown, with error bars representing SD of duplicate wells.

Stability of natalizumab binding under harsh conditions was assessed by exposing natalizumab-spiked serum samples to heat for various lengths of time. The reducing agent GSH was used to simulate *in vivo* natalizumab arm exchange as reported elsewhere (Labrijn *et al*. 2009). Split GSH and non-GSH treated natalizumab-spiked serum samples were incubated at 60 °C for 30 m, 1 h, or 2 h. Control samples were held at 4 °C. Functional binding of the heated natalizumab was assessed by incubating the heat exposed samples with JeKo-1 cells. Cell-bound natalizumab was detected by fluorescently labeled secondary antibody and quantified by flow cytometry. Decreased cell binding was observed with increased heat exposure for both GSH and non-GSH treated samples (Fig. 3a). The heat exposed samples were also assayed by Ntz-01 peptide ELISA and anti-Id ELISA (Fig. 3b). Decreased binding was observed with increased heat exposure by Ntz-01 peptide ELISA, particularly for the GSH treated samples. Changes in binding with heat exposure were not observed by anti-Id ELISA for either GSH or non-GSH treated samples.

**Figure 4:**
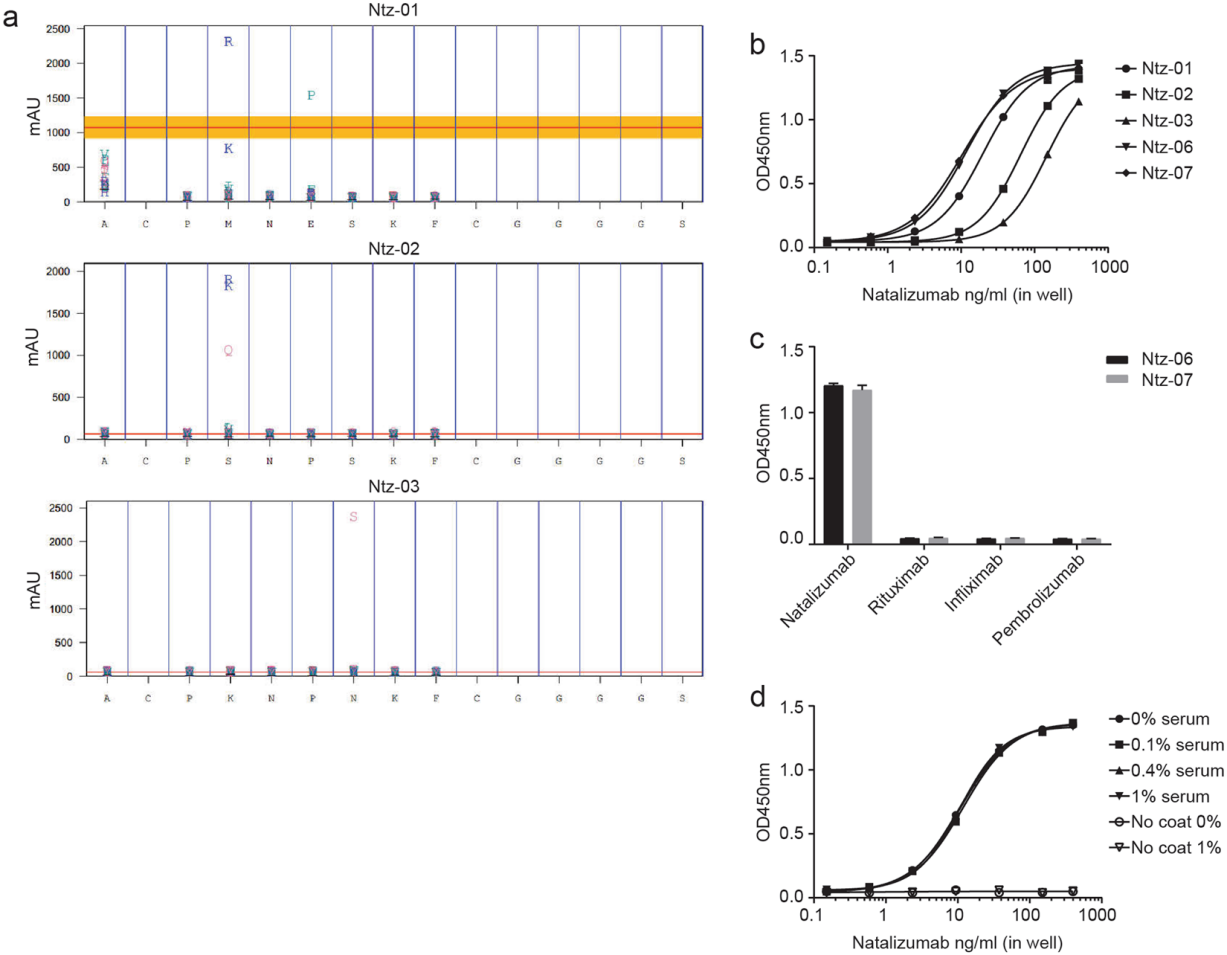
Affinity maturation of selected peptides and performance in ELISA (**a**) Relative binding (arbitrary units) of single amino acid substitution and deletion variations of Ntz-01, Ntz-02, and Ntz-03. Natalizumab binding (y-axis) to each sequence variation is depicted as single-letter amino acid substitutions at each sequence position (x-axis). Deletions are denoted as “−” on the graph (all deletions produce binding similar to or worse than baseline). The red lines indicate natalizumab binding to the parent sequence. (**b**) Natalizumab capture by Ntz-06 and Ntz-07 compared with Ntz-01, Ntz-02, and Ntz-03 in ELISA. (**c**) Selectivity of Ntz-06 and Ntz-07 against control therapeutic mAbs in ELISA. (**d**) Ntz-07 capture of natalizumab spiked into 0.1–1% human serum. For (**b–d**), one representative experiment is shown, with error bars representing SD of duplicate wells.

To identify high affinity motifs, Ntz-01, Ntz-02, and Ntz-03 underwent affinity maturation mutagenesis. Single amino acid substitutions and deletions were done at each random position of the three peptides while maintaining the disulfide constraint. Substitutions were scanned across 18 of the 20 natural amino acids, excluding methionine and cysteine. All peptide variants were tested for natalizumab binding. Linear versions of the parent sequences were also tested but did not bind natalizumab (data not shown). Substitution and deletion results for each sequence are graphically represented in Fig. 4a. The x-axis represents the parent amino acid sequence and the y-axis represents natalizumab binding in arbitrary ELISA units (mAU). Substitutions and deletions at each residue are plotted to indicate natalizumab binding for that substitution (single-letter amino acid nomenclature). The red line indicates natalizumab binding to the parent peptide. Sequence motifs PKNPSKF, PRNPSKF, PQNPSKF, and PRNESKF showed increased binding relative to the parent sequences. From this data, two peptides, Ntz-06 and Ntz-07, were synthesized (Table 3) and assessed as capture reagents in ELISA. Both peptides bound natalizumab with higher apparent affinities than the original peptides (Fig. 4b). Ntz-06 was improved 1.7-fold over Ntz-01. Ntz-07 was improved 14-fold and 6.2-fold over Ntz-03 and Ntz-02, respectively. Both Ntz-06 and Ntz-07 demonstrated specificity when screened against three control mAb therapeutics (Fig. 4c). Ntz-07 was additionally synthesized with C-terminal PEG6 spacer between the GGG linker and the Lys(biotin) to determine if proximity to the plate affected binding efficacy (Online Resource 1, Supplemental Fig. 2). There was no difference in apparent affinity observed between Ntz-07 and the PEGylated variant. Ntz-07 was also assessed in 0.1–1% serum (Fig. 4d). No matrix effects were observed in this serum concentration range. Ntz-07 inhibition of natalizumab capture by the neutralizing anti-Id was also confirmed (data not shown). Thus, the affinity gain observed with Ntz-07 did not disrupt binding at the active site or mitigate binding specificity. Finally, the binding kinetics of all synthesized peptides (Ntz-01–07) were determined by biolayer interferometry (BLI; Table 4). This data mirrored ELISA data, with K_D_ for Ntz-07 of 3.24 nM, compared to 32.1 nM for Ntz-02, and 31.4 nM for Ntz-03.

**Table 3:** Affinity matured peptide sequences. All peptides were synthesized with C-terminal GGG-Lys(biotin).

Name	Sequence	Parent
Ntz-06	ACPRNESKFC	Ntz-01
Ntz-07	ACPKNPSKFC	Ntz-02, Ntz-03

**Table 4:** Apparent affinity data of synthesized peptides/natalizumab as measured by biolayer interferometry (BLI).

Name	K_D_	k_on_	k_dis_	R^2^
Ntz-01	2.91E − 08	1.05E + 05	3.05E − 03	0.9902
Ntz-02	3.21E − 08	1.68E + 05	5.38E − 03	0.9890
Ntz-03	3.14E − 08	2.90E + 05	9.11E − 03	0.9846
Ntz-04	6.03E − 09	1.31E + 05	7.88E − 04	0.9666
Ntz-05	1.05E − 08	1.95E + 05	2.04E − 03	0.9752
Ntz-06	6.87E − 09	2.37E + 05	1.63E − 03	0.9685
Ntz-07	3.24E − 09	1.71E + 05	5.53E − 04	0.9784

## Discussion

This work demonstrates the identification of peptide mimotopes that selectively bind the therapeutic mAb natalizumab. The peptide sequence PKNPSKF is identified as a novel binding motif for natalizumab, and individual peptides containing variations of this motif successfully act as capture reagents for natalizumab in ELISA. This motif does not share sequence homology with natalizumab’s target, α4 integrin (MEME-Suite^18^, data not shown). Of note, four key residues have been identified in α4 integrin that are critical for natalizumab binding, Q152, K201, K208, and K256^19–21^. Interestingly, QNQVKF is the sequence 3′-adjacent to K208, which does bear similarity to the identified motif in this manuscript (Ntz-04, Ntz-05, and Ntz-08 all contain QNXXKF). Further structural analysis is required to compare the conformation of the identified motif to this α4 integrin epitope.

Affinity maturation by single amino acid mutagenesis further confirmed the consensus motif and produced peptides with increased binding performance in ELISA. Similar identification of peptide mimotopes and consensus motifs have been previously observed in a number of systems and applications. Diverse peptide libraries have been used to profile IgE epitopes for allergy diagnostic and therapeutic development, to identify carbohydrate-mimicking peptide sequences for vaccine and therapy discovery, and to discover disease-specific antibody biomarkers with no prior knowledge of disease mechanism through consensus recognition epitopes^22–25^. The current work specifically highlights the application of peptide mimotope ligand identification to biologic therapeutics, and the use of these mimotope ligands (Veritopes^TM^) as *in vitro* assay reagents.

A peptide containing the identified motif competed with the natural ligand (α4 integrin) for natalizumab binding, indicating that the identified Veritopes^TM^ interact with the antigen-binding site of natalizumab. Additionally, the peptide also inhibited natalizumab capture by neutralizing anti-Id in ELISA, further confirming the antigen-binding site as the Veritope^TM^ binding epitope. This is not unexpected; previous work has shown that when libraries of short peptides, 7 to 12 amino acids long, are screened against a purified antibody, the selected peptides almost invariably bind to the antigen-binding site of the antibody and are competed by the natural ligand^17^.

When the binding stability of heat exposed natalizumab was assayed, results by peptide ELISA closely reflected the functional binding results obtained by cell-based assay. Conversely, the results by anti-Id ELISA showed no change across the range of heat exposure times and therefore did not reflect the observed loss in functional binding. This may indicate that Veritopes^TM^, by virtue of being surrogate ligands for the target antibody, are more sensitive to stress-induced conformational changes in the antigen-binding site than anti-idiotype capture reagents.

Peptides have many known advantages as reagents in biological assays. As purely synthetic agents, they are highly stable, modular, inexpensive and reproducible to manufacture, and usable in a variety of assay formats. Peptides specific for rituximab and alemtuzumab have previously been used to measure serum drug levels in CLL patients via ELISA^26^. In addition to these benefits, Veritopes^TM^ may also offer additional specificity for biologic molecules with intact antigen binding pockets. This could have implications for clinical assays using Veritopes^TM^ to quantify active natalizumab in serum for dose monitoring. In this work, natalizumab concentrations assayed in 1% serum reflect the neat serum concentration range 0.6–40 µg/mL, which spans clinically relevant trough concentrations of natalizumab^13–16^.

## Methods

### Antibodies

Natalizumab, rituximab, infliximab, and pembrolizumab were obtained from the UCSD Moores Cancer Center Pharmacy.

### Biopanning

Four phage display libraries [custom in-house or New England Biolabs (NEB), Ipswich MA] were used (Table 1). Wells of a Costar 96-well high binding plate (Corning Life Sciences, Tewksbury MA) were coated with 10 µg/ml of natalizumab in tris buffered saline (TBS, 20 mM Tris-HCl, 150 mM NaCl, pH 7.4, diluted from 20X, Teknova, Hollister CA) overnight at 4 °C, followed by blocking for 1 h at room temperature (RT). Wells were blocked with 5% bovine serum albumin fraction V/TBS (BSA, Thermo Fisher, Waltham MA) for Libraries 1–3, and with Blocker™ Casein in TBS (Thermo Fisher) for Library 4. Wells were washed three times with TBS, and phage libraries (10^11^ phages/100 µl) diluted in the respective blocking buffers were added and incubated for 2 h at RT. Wells were washed six times with TBS. Remaining bound phages were eluted with 0.1 M glycine-HCl pH 2 for 10 m and then neutralized with 1 M Tris pH 8.5 (Teknova). The eluted phages were amplified by infecting ER2738 *E. coli* (NEB) and then titered on LB/IPTG/Xgal plates for subsequent rounds of selection. Three rounds of biopanning were performed. In round two of the selection, 10 total TBS washes were performed to remove non-specific phage. In round three, 10 total washes with TBS 0.05% Tween-20 (TBST; Tween-20, Thermo Fisher) were performed. Selected phage clones were isolated and their inserts sequenced using the −96gpIII primer (NEB).

### Confirmation of Phage Clone Binding

Isolated phage clonal populations were incubated with natalizumab- or control mAb-coated wells under the conditions used during round three of library panning. Bound phages were eluted and titered. Phage clones with positive titers against natalizumab compared to the control mAb were selected for peptide synthesis.

### Peptide Synthesis

All peptides were synthesized as C-terminal free acids and N-terminal free amines with an additional C-terminal GGG-Lys(biotin) for assay immobilization (Genscript, Piscataway NJ, CPC Scientific, Sunnyvale CA, or Abbiotec, San Diego CA).

### Peptide ELISA

Peptide ELISAs were performed using two protocols. Protocol-1 was used for Figs 1c,e, 2b and 3b. Protocol-2 was used for Figs 1d and 4b–d, and Online Resource 1 (Supplemental Fig. 2).

All incubations were conducted at RT on an orbital shaker unless otherwise noted. All washes were performed using an automated plate washer (Columbus Pro, Tecan, Durham NC).

#### Protocol-1

Wells of a NeutrAvidin-coated 96-well plate or 8-well strips (Thermo Fisher) were coated with 100 µl of biotinylated peptide at 7.5 µg/ml in molecular grade H_2_O (GE Healthcare Life Sciences, Logan, UT) for 1 h. Wells were washed five times with TBST. Wells were then blocked with 200 µl of 5% normal goat serum diluted from 10% normal goat serum (Thermo Fisher) in TBS for 1 h. Wells were washed five times with TBST. Antibody was spiked into 2.5% BSA in TBST (2.5% BSA/TBST) or into pooled defibrinated human AB serum (serum; Gemini, Woodland CA) diluted in 2.5% BSA/TBST. The final dilution of serum was 0.4% unless otherwise noted. Wells were incubated with 100 µl of antibody-spiked samples for 1 h and then washed five times with TBST. Bound natalizumab was detected with mouse anti-human IgG4 horse radish peroxidase (anti-IgG4-HRP; Abcam, Cambridge, MA) diluted 1:2000-1:5000 in TBST. Wells were incubated with 100 µl of diluted anti-IgG4-HRP for 30 m and then washed nine times with TBST. Turbo TMB substrate (Thermo Fisher) was added to the wells and allowed to develop for 12–15 m shielded from light. The reaction was stopped with 1 M H_2_SO_4_ (Thermo Fisher). Optical density (OD) was read at 450 nm on a Multiskan FC plate reader (Thermo Fisher) and data were analyzed with GraphPad Prism v5 or v7 software (Graphpad Software Inc, La Jolla CA).

#### Protocol-2

Wells of a streptavidin-coated 96-well plate or 8-well strips (Thermo Fisher) were coated with 100 µl of peptide in TBS at 1 µg/ml for 1 h and washed five times with TBST. Wells were then blocked with 200 µl of 2.5% BSA/TBS for 1 h and washed five times with TBST. Antibody was spiked into 2.5% BSA/1X casein (diluted from 5X, Surmodics, Eden Prarie MN) in TBST or into serum diluted in 2.5% BSA/1X casein/TBST. The final dilution of serum was 0.4% unless otherwise noted. Wells were incubated with 100 µl of antibody-spiked samples for 1 h and then washed five times with TBST. Bound natalizumab was detected with anti-IgG4-HRP diluted 1:5000 in 2.5% BSA/TBST. Wells were incubated with 100 µl of diluted anti-IgG4-HRP for 30 m and then washed nine times with TBST. Turbo TMB substrate was added to the wells and allowed to develop for 12–15 m shielded from light. The reaction was stopped with 1 M H_2_SO_4_. OD was read at 450 nm and data were analyzed as in Protocol-1.

### Anti-Idiotype ELISA

Wells of a Costar 96-well high binding plate were coated with 100 µl of natalizumab neutralizing anti-Idiotype (anti-Id) Fab fragment (Bio-Rad, Hercules CA) in phosphate-buffered saline (PBS; Corning Life Sciences) at 1 µg/ml overnight at 4 °C. The remining protocol proceeded as in peptide ELISA Protocol-1, with the following exceptions. 1) PBS/PBST was used in place of TBS/TBST (except for washes), 2) goat anti-human IgG HRP (Jackson Immunoresearch, West Grove PA) diluted 1:5000 was used as the secondary conjugate, and 3) TMB substrate was allowed to develop for 8–10 m.

### Peptide Competition ELISA

The Anti-Idiotype ELISA protocol was used. Peptide was incubated with natalizumab-spiked samples in solution for 1 h prior to sample addition.

### CD49d+ Cell Based Peptide Competition Assay

Ntz-01 or control peptide was incubated with natalizumab in 5% fetal bovine serum (FBS, GE Healthcare Life Sciences) in Dulbecco’s phosphate-buffered saline (DPBS, Corning Life Sciences) with 0.05% NaN_3_ (Thermo Fisher) for 1 h on ice. CD49d+ JeKo-1 cells were suspended in 5% FBS/DPBS/0.05% NaN_3_, and 25 µl of suspension (10^5^ cells) were added to 25 µl of sample and incubated for 20 m on ice. Cells were washed two times with 5% FBS/DPBS/0.05% NaN_3_ and spun down at 300xg for 5 m at 4 °C. Cells were then resuspended in 100 µl of 5% FBS/DPBS/0.05% NaN_3_ containing a 1:200 dilution of mouse anti-human IgG4 Fc Alexa Fluor 647 (Southern Biotech, Birmingham AL) and incubated for 20 m on ice. Cells were washed two times with 5% FBS/DPBS/0.05% NaN_3_, resuspended in 150 µl 1% paraformaldehyde (PFA, Thermo Fisher) in PBS, collected on flow cytometer (BD FACSCalibur, BD Biosciences, San Jose CA), and analyzed with FlowJo software v7.6.5 (BD Biosciences subsidiary, Ashland OR).

### CD49d+ Cell Based Binding Assay

Natalizumab-spiked samples in serum were diluted 25-fold in 5% FBS/DPBS/0.05% NaN_3_. CD49d+ JeKo-1 cells were suspended in 5% FBS/DPBS/0.05% NaN_3_, and 25 µl of suspension (10^5^ cells) were added to 25 µl of diluted sample and incubated for 20 m on ice. Washes, secondary incubation, collection, and analysis proceeded as in the CD49d+ Cell Based Peptide Competition Assay protocol.

### GSH-treatment of Natalizumab

Natalizumab was spiked into neat serum. The samples were split in half and 100 mM Glutathione reduced (GSH, Sigma-Aldrich, St. Louis MO) in H_2_O was added to one set to produce a final concentration of 3 mM GSH. Samples were then incubated overnight at 37 °C to induce arm exchange of IgG4 molecules. For ELISA assays, samples were subsequently diluted 250-fold in 2.5% BSA/TBST to produce a final serum concentration of 0.4%.

### Heat Exposure of Natalizumab

Samples containing GSH treated or non-GSH treated natalizumab in serum were heated at 60 °C for 30 m, 1 h, or 2 h. Control samples were held at 4 °C. For ELISA assays, samples were subsequently diluted 250-fold in 2.5% BSA/TBST to produce a final serum concentration of 0.4%.

### Peptide Affinity Maturation

Affinity maturation by amino acid mutagenesis was performed by Pepscan (Lelystad, Netherlands). Briefly, a library of peptides was synthesized on an amino functionalized polypropylene support using solid-phase Fmoc synthesis. The library was constructed from single point substitutions and deletions of each position in the parent peptide sequences. Eighteen of the 20 natural amino acids, excluding methionine and cysteine, were used for substitutions. Synthesis protocols were performed by custom modified JANUS liquid handling stations (Perkin Elmer, Waltham MA).

Natalizumab binding to the library peptides was tested by array-based ELISA (Pepscan). Briefly, peptide arrays were incubated with natalizumab solution overnight at 4 °C. After washing, the arrays were incubated with a 1:1000 dilution of peroxidase-labeled anti-human IgG secondary antibody for 1 h at 25 °C. After washing, the peroxidase substrate 2,2′-azino-di-3-ethylbenzthiazoline sulfonate (ABTS) and 20 μl/ml of 3% H_2_O_2_ were added for 1 h. Color development was measured and quantified by charge coupled device (CCD) camera and an image processing system. The values obtained from the CCD camera range from 0 to 3000 milli-Arbitrary Units (mAU).

### Biolayer Interferometry (BLI)

To determine binding constants for association and dissociation of natalizumab with Ntz-01–07, BLI was performed by Antibody Solutions (Mountain View CA), using an Octet Red96 (Pall ForteBio LLC, Fremont CA). Briefly, 10 µg/ml of peptide was immobilized onto a streptavidin sensor. Three-fold dilutions of natalizumab were in solution, at concentrations from 1–600 nM (7 total dilutions). The assay steps were: sensor check (30 s), load antibody (600 s), baseline (300 s), antibody association (550 s), antibody dissociation (550 s). TBS was used as the buffer.

### Statistics

ELISA experiments were performed with duplicate wells and results are reported with standard deviation (SD) of these wells. Experiments were performed 2–3 times, and one representative experiment is shown in the Figures. In some cases, samples were divided in replicate experiments (e.g. for selectivity replicate, Ntz-01–03 was performed on one plate and Ntz-06–07 was performed on a separate plate).

## Electronic supplementary material

Supplementary Information

## Data Availability

All data generated or analyzed during this study are included in this published article (and its Supplementary Information files).
