# Supplementary material for: Identification of Peptide Mimotope Ligands for Natalizumab
Source: Sci Rep. 2018 Sep 27;8:14473. doi: 10.1038/s41598-018-32832-1 (PMC6160459; doi:10.1038/s41598-018-32832-1)
Supplement: Supplementary file 1 — Supplementary Information [file 41598_2018_32832_MOESM1_ESM.pdf]

Identification of Peptide Mimotope Ligands for Natalizumab

Laura E. Ruff, Jessica A. Pfeilsticker, Nicholas E. Johnsen, Sarah Nocchi, and Bradley T. Messmer*
Abreos Biosciences, 3550 General Atomics Ct, Bldg G02, Rm 559, San Diego CA 92121
*Corresponding Author, bmessmer@abreosbio.com, 858-248-9253

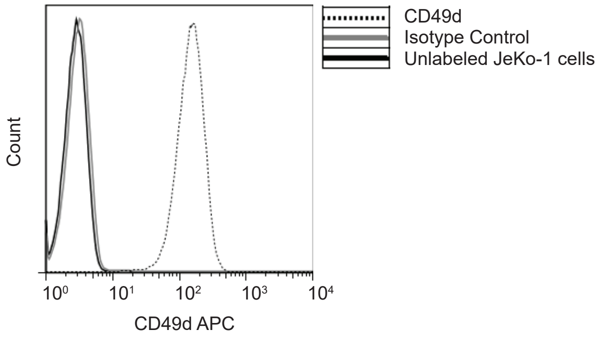

Supplemental Fig. 1 Confirmation of JeKo-1 cell CD49d (α4 integrin) expression

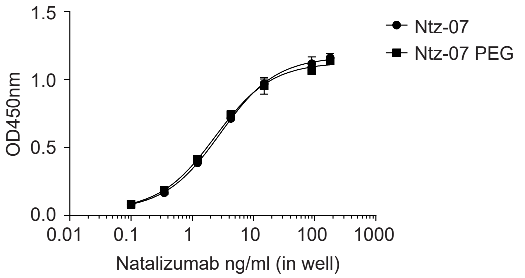

Supplemental Fig. 2 Natalizumab capture with Ntz-07 synthesized with a PEG6 linker between the main sequence and C-terminal Lys(biotin)
